# P-1707. Rapid Detection of Extended-Spectrum Beta-Lactamase-Producing Bacteria Using MBT STAR-Cepha® Technology

**DOI:** 10.1093/ofid/ofaf695.1879

**Published:** 2026-01-11

**Authors:** Victoria Petty, Brent D Nelson, Ashley Rogers, Heather Fillmore, Bert K Lopansri

**Affiliations:** Intermountain Health, Murray, UT; University of Utah, Salt Lake City, UT; Intermountain Medical Center, Murray, Utah; Intermountain Medical Center, Murray, Utah; Intermountain Healthcare, Salt Lake City, Utah

## Abstract

**Background:**

Rapid identification of antibiotic resistance is critical to improve antibiotic use and outcomes in patients infected with extended-spectrum beta-lactamase (ESBL) harboring Enterobacteriaceae. Commercially available molecular methods to detect the presence of ESBL are costly and only detect *bla*_CTX-M_. Faster and more complete results at a lower cost may be attainable with matrix-assisted laser desorption ionization time-of-flight mass spectrometry (MALDI-TOF).

**Figure:**
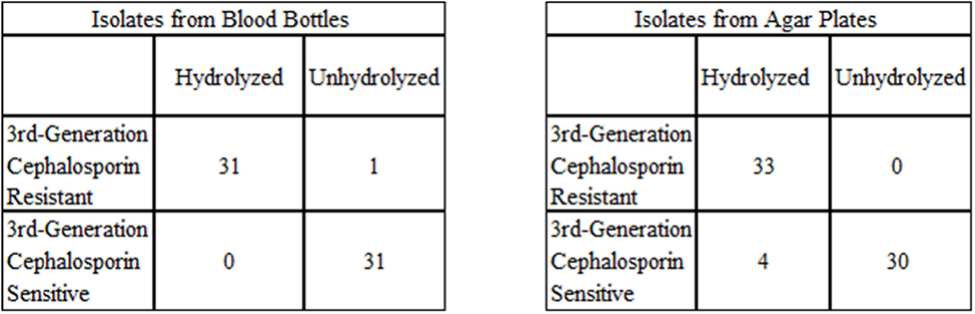
STAR-Cepha Sensitivity and Specificity

**Methods:**

We conducted a clinical evaluation of the MBT STAR-Cepha® kit (Bruker) using clinical isolates confirmed phenotypically to be resistant to third generation cephalosporins. Gram-negative bacterial isolates were taken from cryopreserved and contemporary clinical samples. Tested isolates were inoculated into blood culture broth and plated onto solid media, and both sample types were tested with the Star Cepha® kit in accordance with the manufacturer’s recommendations. The spectra undergo automated data analysis to detect a mass shift of the antibiotic. In the case of ESBL-producing bacteria, multiple product peaks are detected due to the hydrolyzation of the antibiotic by enzymatic activity. A single peak of the unhydrolyzed antibiotic is present in the case of non-ESBL-producing bacteria.

**Results:**

Among the 71 isolates tested (68 blood culture broth, 71 solid media), 46 were *E. coli*, 13 *Klebsiella pneumoniae*, 6 *Klebsiella oxytoca*, 5 *Proteus mirabilis*, and 1 *Serratia marcescens*. Of these, 34 were determined to be resistant to 3^rd^ generation cephalosporins and 37 susceptible by standard methods. MALDI correctly identified the presence of ESBL in 31/34 broth cultures and 33/34 from colonies (Table). Broth testing yielded a sensitivity of 97% and specificity of 100% with 5 indeterminate results (2 ESBLs and 3 non-ESBLs). Isolates from agar plates yielded a sensitivity of 100% and a specificity of 88% with 4 indeterminate results (1 ESBL and 3 non-ESBLs).

**Conclusion:**

Star-Cepha® is a promising tool to rapidly identify ESBL harboring Enterobacteriaceae directly from positive blood cultures and isolated colonies in solid media. Further analysis is needed to expand the data pool and investigate how the different ESBL genotypes interact with the assay.

**Disclosures:**

Bert K. Lopansri, MD, D(ABMM), FIDSA, Karius: Advisor/Consultant|Seegene: Advisor/Consultant

